# New Insights of the Zn(II)-Induced P2 × 4R Positive Allosteric Modulation: Role of Head Receptor Domain SS2/SS3, E160 and D170

**DOI:** 10.3390/ijms21186940

**Published:** 2020-09-22

**Authors:** Francisco Andrés Peralta, J. Pablo Huidobro-Toro

**Affiliations:** 1 Laboratorio de Farmacología de Nucleótidos, Departamento de Biología, Facultad de Química y Biología, Universidad de Santiago de Chile, Santiago 22434, Chile; francisco.andres.peralta@gmail.com; 2 Centro para el Desarrollo de la Nanociencia y la Nanotecnología (CEDENNA), Santiago 22434, Chile

**Keywords:** P2 × 4R, Zn(II) allosteric modulation, P2 × 4R SS2/SS3 microenvironment, head receptor domain acid residues, P2 × 4R electrostatic potential

## Abstract

P2 × 4R is allosterically modulated by Zn(II), and despite the efforts to understand the mechanism, there is not a consensus proposal; C132 is a critical amino acid for the Zn(II) modulation, and this residue is located in the receptor head domain, forming disulfide SS3. To ascertain the role of the SS2/SS3 microenvironment on the rP2 × 4R Zn(II)-induced allosteric modulation, we investigated the contribution of each individual SS2/SS3 cysteine plus carboxylic acid residues E118, E160, and D170, located in the immediate vicinity of the SS2/SS3 disulfide bonds. To this aim, we combined electrophysiological recordings with protein chemical alkylation using thiol reagents such as N-ethylmaleimide or iodoacetamide, and a mutation of key amino acid residues together with P2 × 4 receptor bioinformatics. P2 × 4R alkylation in the presence of the metal obliterated the allosteric modulation, a finding supported by the site-directed mutagenesis of C132 and C149 by a corresponding alanine. In addition, while E118Q was sensitive to Zn(II) modulation, the wild type receptor, mutants E160Q and D170N, were not, suggesting that these acid residues participate in the modulatory mechanism. Poisson–Boltzmann analysis indicated that the E160Q and D170N mutants showed a shift towards more positive electrostatic potential in the SS2/SS3 microenvironment. Present results highlight the role of C132 and C149 as putative Zn(II) ligands; in addition, we infer that acid residues E160 and D170 play a role attracting Zn(II) to the head receptor domain.

## 1. Introduction

Zn(II) is a trace metal essential to life; it is relevant both as a structural protein element and as a catalyst in several enzymes such as superoxide dismutase [1] or as lyases or hydrolases in metabolic pathways [2]. Zn(II) also plays a role in transcription factors as the classic Zn(II)-fingers [3]. The metal is also a ZAC [4], TRPA1 [5], and Slo1(BK) [6] ion channel agonist as reviewed recently [7]. In proteins, sulfhydryl, imidazole, or carboxyl side groups are the predominant Zn(II) coordination ligands; consistently, the metal stabilizes protein structure, through its preferential coordination with Cysteine (C), Histidine (H), Aspartate (D), or Glutamate (E) residues. The intracellular concentration of this transition metal is highly regulated; it is transported into cells by a set of multiple ZIP transporters while a different family of ZnT transporters govern its intracellular trafficking [8,9,10,11].

In the central nervous system, Zn(II) is mobilized into synaptic vesicles through ZnT3 transporters [12,13]; the metal is co-stored and co-released to the synapsis with glutamate [14,15]. Zn(II) is also known to modulate ion channels relevant to brain transmission and cell physiology [7,16,17]; it either modifies the number of membrane channels as in ENaC [18], or the open probability as in NMDA [19], or the unitary conductance as in PIEZO1 [20]. Our laboratory and others, described that Zn(II) acts as a positive allosteric modulator of ATP-activated ion channels (P2 × Rs) [21,22,23,24], as evidenced by a 5-fold leftward displacement of the P2 × 2R or P2 × 4R ATP concentration–response curves, suggesting that the metal binding to P2 × Rs increases ATP affinity. Moreover, Zn(II) enhanced long-term potentiation in the brain CA1 region, a mechanism related to the synaptic P2 × R modulator role [25].

Regarding the role of Zn(II) as an allosteric modulator, a challenging issue of our keen interest is to localize its putative allosteric site to fully understand the molecular basis of the metal-induced P2 × 4R allosteric regulation. Based on the rapid ATP-gated current potentiation, plus the finding that the metal does not require preapplication [21] to consistently elicit a leftward displacement of the ATP concentration response curve [26], we inferred that the Zn(II) binding site must be easily accessible and postulated that it might be localized in the extracellular P2 × 4R domain to likely either augment the ATP affinity at its orthosteric site or facilitate the open probability or conductance. In the search for putative Zn(II) coordination sites, and fully aware that the metal coordinates preferentially with free sulfhydryl, imidazole, or carboxylic acid residues, we examined putative metal coordination sites in the extracellular head P2 × 4R domain. Its crystal structure revealed five conserved SS bonds common to all P2 × Rs; three of these are found in the extracellular head receptor domain [27,28]; the SS2 bond is formed by C126 and C149, the SS3 bond is formed by C132 and C159. The P2 × 4R contains only three histidine imidazoles in its extracellular domain sites, one of which is associated to Cu(II) allosteric inhibition rather than Zn (II) potentiation [29], an argument that weakens the eventual role of these residues as putative Zn(II) ligands.

In this investigation, we focused on free thiols in the extracellular P2 × 4R head domain as putative metal coordination ligands, since prior studies from our group and others showed that C132 is critical for the Zn(II) positive modulation [30,31]. This result allowed us, in principle, to suppose that C132 might act as one of the Zn(II) ligands in the P2 × 4R head domain. Interestingly, C126, a residue close to C132, does not participate on the Zn(II) allosteric modulation [31,32]. Free carboxylic residues may also play a role in Zn(II) coordination; some of them were examined previously, such as D129, D131, and D138 [30]; the mutation of none of these residues abolished the Zn(II) positive allosteric modulation, allowing us to discard the participation of these carboxylic acid residues as additional metal ligands. Since C132 is involved in the SS3 bond, we deduced that for Zn(II) to coordinate with this residue thiol, there must be a fraction of reduced SS3 or alternatively that the metal in this tridimensional environment displaces the redox equilibrium of these disulfides making free thiols available susceptible to alkylation. A similar argument must be raised for the SS2 bond, since the metal requires at least four coordination ligands, two of which might be based on cysteine thiols derived from the SS2/SS3 bonds in the P2 × 4R head domain. Following this reasoning, we now examined, as part of our strategy, the presence of free thiols susceptible to chemical alkylation. Our working hypothesis predicts that the alkylation should only occur upon P2 × 4R co-incubation with alkylators plus Zn(II), providing as a control protocols, the incubation of thiol reagents in the metal’s absence.

The present report summarizes our progress addressed by combining electrophysiological recordings of wild type (WT) P2 × 4R incubated with thiol reagents in the presence or absence of Zn(II). To further confirm the studies using chemical protein modifications, site-directed P2 × 4Rs mutants of discrete P2 × 4R residues were also examined. Finally, using bioinformatic tools the Poisson–Boltzmann equation was solved for WT and acid residue mutants with the aim of assessing changes in the external head domain electrostatic potential.

## 2. Results

### 2.1. NEM Alkylation of Free P2 × 4R Thiols Is Dependent on Zn(II)

#### 2.1.1. Zn(II) Allosteric P2 × 4R Modulation

**Figure 1:**
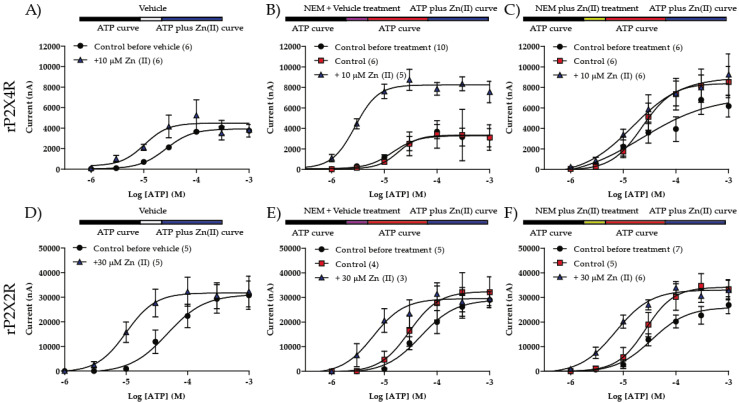
NEM treatment in presence of Zn(II) abolish metal modulation in rP2 × 4R but not on rP2 × 2R. The protocol consist on a full ATP concentration–response curve (black bar in protocols and black filled circles in graphs) followed by a 5 min treatment (vehicle = white, NEM + vehicle = purple, NEM + Zn(II) = yellow) and 20 min washout and finally a full ATP concentration–response curve in absence (red bar in the protocols and red filled squares in graphs) or presence of Zn(II) (blue bar in the protocols and blue filled triangles on graphs). (**A**) Vehicle treatment on rP2 × 4R, (**B**) NEM treatment on rP2 × 4R, (**C**) NEM + Zn(II) treatment on rP2 × 4R, (**D**) vehicle treatment on rP2 × 2R, (**E**) NEM treatment on rP2 × 2R and (**F**) NEM + Zn(II) treatment on rP2 × 2R. Circles, squares and triangles represent ± SEM. N is indicated in parenthesis on each graph.

Prior to examining P2 × 4R alkylation, we quantified the Zn(II)-induced allosteric modulation. In the group of vehicle-treated oocytes (Barth’s buffer), 10 µM Zn(II) reduced the ATP EC_50_ 3.82-fold from 35.3 ± 6.6 to 9.2 ± 2.7 µM (*p* = 0.016, *n* = 6, Figure 1A), but not the nH (1.4 ± 0.2 v/s 1.4 ± 0.2, *p* = 0.16, *n* = 6), nor I_max_ (4.1 ± 0.6 v/s 4.8 ±1.2 µA, *p* = 0.28, *n* = 6). The Zn(II) concentration required for the potentiation of the ATP-evoked currents ranged between 10 and 100 µM (Figure S1, left), values within those previously reported [21,24,26]. The metal effect was reversible since after 10 min washout, the initial 10 µM ATP-gated current was fully recovered. No ATP-evoked currents where observed in non-injected oocytes.

#### 2.1.2. P2 × 4R NEM Alkylation Required Zn(II) to Annul Metal-Induced Modulation

To examine whether NEM alkylated free P2 × 4R thiols, and therefore abolished the Zn(II)-induced-potentiation, oocytes were incubated with 300 µM NEM for 5 min in two independent, matched, protocols applying (1) NEM + Vehicle and (2) NEM + Zn(II). In the control, NEM plus vehicle treatment, Zn(II) displaced the concentration—response (Figure 1B and Table 1) and also increased efficacy. Following NEM treatment plus 10 µM Zn(II), the metal failed to evidence the Zn(II)-induced modulation (Figure 1C and Table 1). NEM treatment did not augment I_max_, nor did it modify the P2 × 4R potency (Table 1), an indication that this chemical did not alter the receptor ATP affinity. The irreversible nature of the alkylation was demonstrated because the lack of metal potentiation endured at least 1 h after NEM treatment.

#### 2.1.3. NEM Application Did Not Annul P2 × 2R Zn(II) Modulation with or without Metal Co-Application

As a further control of nonspecific NEM effects on P2 × R or other membrane proteins, the P2 × 2R was used, this receptor is also positively modulated by Zn(II) [33]. The P2 × 2R ATP-evoked currents are potentiated by 30–300 µM Zn(II) (Figure S1, right) but the modulation depends on histidines [34,35] and not on cysteines as postulated for the P2 × 4R [36]. Based on these premises, the P2 × 2R we used as a further NEM treatment control. A total of 30 µM Zn(II) shifted leftwards the ATP concentration–response curve (EC_50_: 56.4 ± 7.4 v/s 10.9 ± 1.3 µM, *p* = 0.008, *n* = 5) without altering nH: (1.7 ± 0.1 v/s 1.9 ± 0.4, *p* = 0.55, *n* = 5) or I_max_: (30.9 ± 5.8 v/s 32.5 ± 5.1 µA, *p* = 0.69, *n* = 5; Figure 1D). After NEM treatment plus a vehicle, co-application of 30 µM Zn(II) shifted the ATP concentration–response to the left (Figure 1E and Table 1). Likewise, following NEM plus Zn(II) treatment, the metal also modulated the P2 × 2R (Figure 1F and Table 1). None of the treatments modified the EC_50_, nH or I_max_ compared to controls before the treatments (Table 1).

Altogether, these results strongly suggest that after NEM treatment with or without Zn(II), no sites sensitive to NEM were found in the P2 × 2R, since the Zn(II)-induced allosteric response was not lost. In addition, based on the P2 × 2R findings, we infer that the likely P2 × 4R residues alkylated by NEM are presumably cysteine, compatible with the working hypothesis and further differentiating P2 × 2R from the P2 × 4R.

### 2.2. P2 × 4R Treatment with Non-Charged Alkylators in Presence of Zn(II) Annuls Metal Potentiation

A simpler and shorter assay to test the Zn(II) potentiation was developed to better assess the loss of Zn(II) modulation elicited by the joint co-treatment of NEM plus Zn(II). It consisted of challenging the oocytes with a single ATP concentration (10 µM) rather than a full ATP concentration–response curve, followed 10 min later by the co-application of ATP plus Zn(II) (10 µM for P2 × 4R and 30 µM for P2 × 2). Ten minutes after alkylator washout, cells were treated with Zn(II) plus NEM or iodoacetamide (IA) for 5 min followed by 20 min of alkylator washout. Next, the P2 × R was tested again to examine the magnitude of the Zn(II) potentiation.

**Figure 2:**
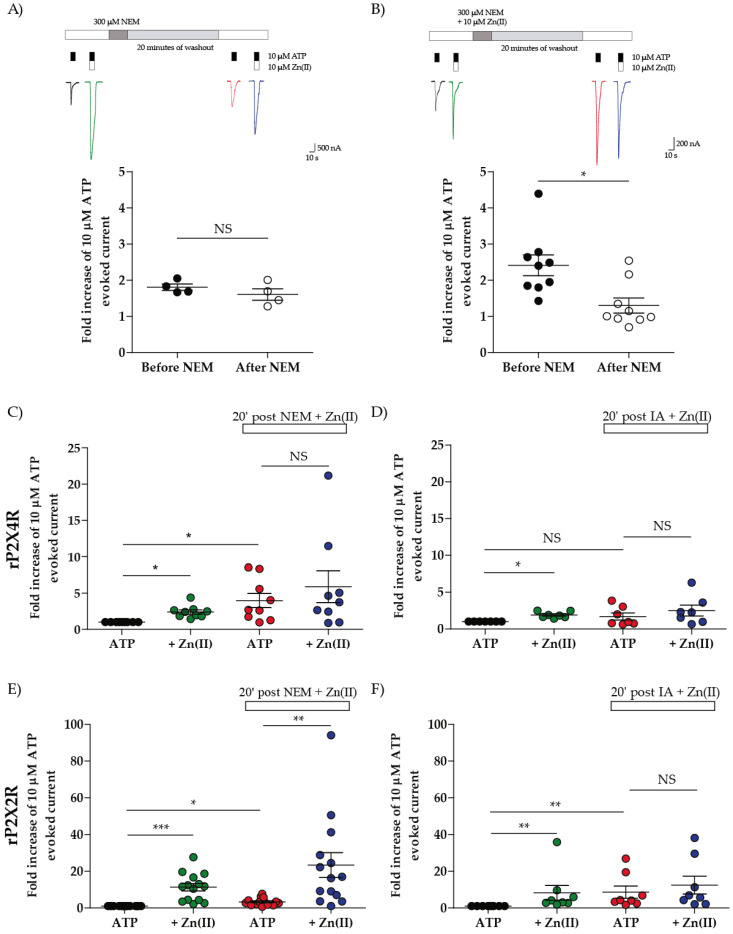
Zn(II) potentiation is annulled in rP2 × 4R by NEM + Zn(II) and IA + Zn(II) treatments but only with IA + Zn(II) treatment in rP2 × 2R. (**A**) representative traces (up) of ATP-evoked currents in absence (black) and presence of Zn(II) (Green) and after NEM treatment (red and blue) and quantification of the fold increase of ATP-evoked currents by Zn(II) (down). (**B**) representative traces (up) of ATP-evoked currents in absence (black) and presence of Zn(II) (Green) and after NEM + Zn(II) treatment (red and blue) and quantification of the fold increase of ATP-evoked currents by Zn(II) (down). * *p* < 0.05, NS = non significative according to the Mann–Whitney test. (**C**,**D**) quantification of the fold increase of first ATP evoked current in NEM + Zn(II) and IA + Zn(II) treatments in rP2 × 4R and (**E**,**F**) quantification of the fold increase of first ATP evoked current in NEM + Zn(II) and IA + Zn(II) treatments in rP2 × 2R. * *p* < 0.05, ** *p* < 0.01, *** *p* < 0.001 and NS = non significative according to the Kruskal–Wallis test.

Using this simplified protocol, as expected for controls, 10 µM Zn(II) potentiated almost 2-fold the 10 µM ATP-evoked currents in P2 × 4R prior to any treatments (compare Figure 2A–D with Figure S1). After NEM treatment and its washout period, ATP-evoked currents were potentiated by Zn(II) as usual prior to treatments (1.6 ± 0.2 v/s 1.8 ± 0.1-fold increase, after v/s before treatment, respectively, *p* = 0.49, *n* = 4; Figure 2A); however, after NEM plus Zn(II) incubation, the ATP-evoked currents were no longer potentiated by Zn(II) (1.3 ± 0.2 v/s 2.4 ± 0.3-fold increase, after v/s before treatment, respectively, *p* = 0.0056, *n* = 9; Figure 2B,C), consonant with the results presented on the previous section (compare Figure 2B with Figure 1C). Interestingly, ATP-evoked currents were significantly increased after NEM plus Zn(II) treatment 4.0 ± 1.0-fold increase (according to a Kruskal–Wallis with Dunns post-test) (Figure 2C). This effect was not observed following NEM or IA treatments in the absence of the metal. The NEM effect lasted for at least an hour since after an additional 40 min, no Zn(II) potentiation was observed (Figure S2), compatible with a covalent modification as anticipated from the alkylation control results shown in the previous section.

Next, NEM was replaced by IA using this same protocol. The ATP-evoked currents after the treatment were not increased, and, as with NEM plus Zn(II), the metal did not potentiate the ATP-evoked currents (Figure 2D) indicating that non-charged cysteine alkylators smaller than 185 g/mol, abolished the Zn(II)-induced potentiation on rP2 × 4R (MW:185 g/mol for IA and 125 g/mol for NEM). Likewise, for the P2 × 2R, 30 µM Zn(II) potentiated 11.4 ± 2.0 and 8.3 ± 4.0 times the ATP-evoked currents, respectively (Figure 2E,F). After both treatments the ATP-evoked currents where enhanced (3.3 ± 0.5 and 8.6 ± 3.3 fold, respectively), but only IA annulled the Zn(II) potentiation (according to Kruskal–Wallis with Dunn’s post-test) (Figure 2F), meaning that the loss of Zn(II) potentiation by NEM plus Zn(II) treatment is P2 × 4R selective.

#### Charged Thiol Alkylators Such as DTNB or IAc Did Not Suppress the Zn(II) Potentiation

**Figure 3:**
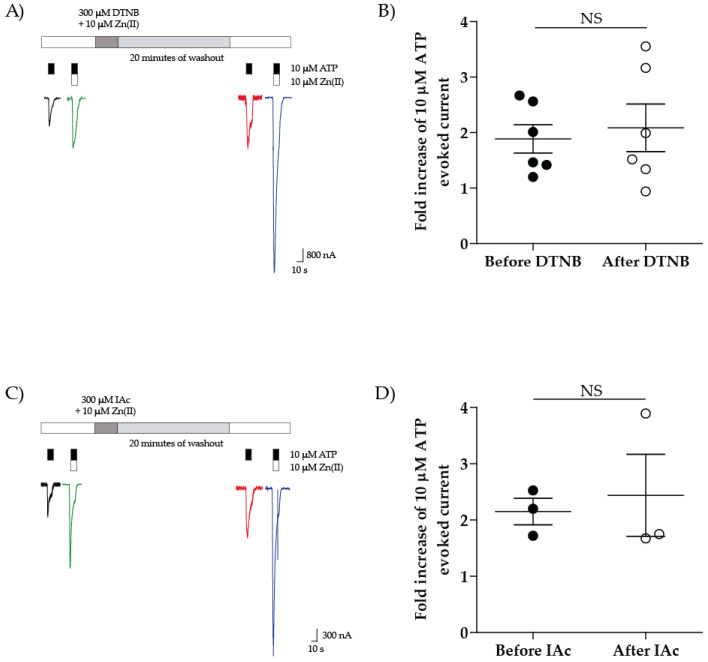
Neither DTNB + Zn(II) nor IAc + Zn(II) treatments abolish the Zn(II) potentiation in rP2 × 4R. (**A**) representative traces of ATP-evoked currents in absence (black) and presence of Zn(II) (Green) and after DTNB + Zn(II) treatment (red and blue) and (**B**) quantification of the fold increase of ATP-evoked currents by Zn(II). (**C**) representative traces of ATP-evoked currents in absence (black) and presence of Zn(II) (Green) and after IAc + Zn(II) treatment (red and blue) and (**D**) quantification of the fold increase of ATP-evoked currents by Zn(II). *N* = 6 for DTNB treatment and 3 for IAc treatment, paired data, NS = non significative according to Mann–Whitney test.

We next replaced NEM for 300 µM 5,5’-dithiobis-(2-nitrobenzoic) acid (DTNB, MW: 396 g/mol) plus 10 µM Zn(II) or by 300 µM iodoacetate (IAc, 186 g/mol) plus 10 µM Zn(II) treatments. The observed results showed that neither of the reagents blocked the P2 × 4R Zn(II)-induced potentiation (2.1 ± 0.4 v/s 1.9 ± 0.3-fold, after v/s before DTNB treatment, respectively, *p* = 0.93, *n* = 6; 2.4 ± 0.7 v/s 2.2 ± 0.2-fold, after v/s before IAc treatment, respectively, *p* = 0.75, *n* = 3; Figure 3), confirming that not only the alkylator size but also the charge is determinant to annul the Zn(II) potentiation. As a proof of concept and in support of the advantage of the short protocol, we performed a complete concentration–response curve by treating oocytes with 300 µM DTNB + 10 µM Zn(II) followed by washout and a new concentration–response curve in the presence of Zn(II). The resultant EC_50_ was not different from the control (79.8 ± 64.4 v/s 50.3 ± 16.0 µM; *p* = 0.35, *n* = 3), but it was consistently reduced in presence of Zn(II) (50.3 ± 16.0 v/s 10.23 ± 5.2 µM, Control v/s 10 µM Zn(II), respectively, *p* = 0.05, *n* = 3; Figure S3). Neither the nH nor the I_max_ was modified by treatments. In sum, contrary to expectations, neither DTNB nor IAc modified the Zn(II)-induced modulation, allowing us to conclude that because of reagent negative charge, neither DTNB or IAc accessed the head receptor domain free thiol site.

### 2.3. DTT Did Not Potentiate ATP-Evoked Currents but Abrogated the NEM Effect on the Zn(II)-Induced Potentiation

**Figure 4:**
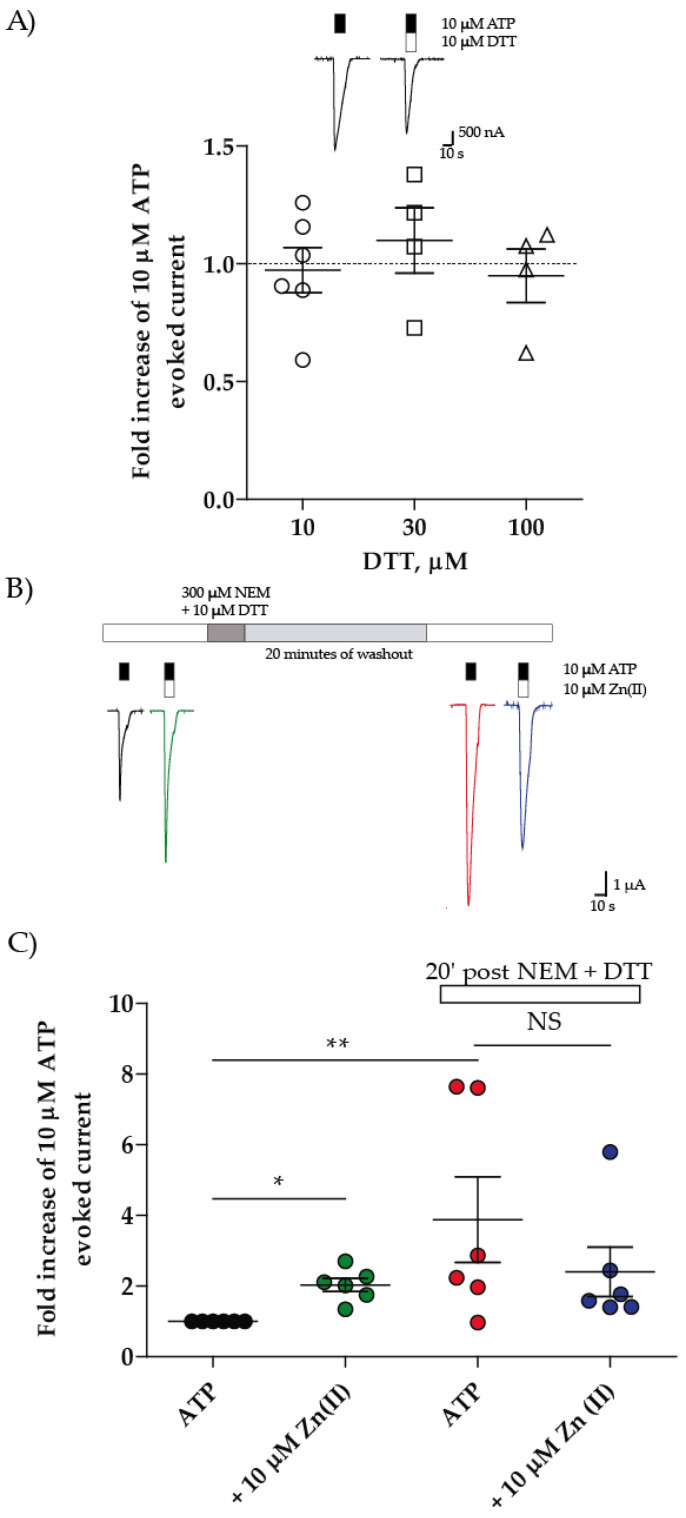
Dithiothreitol (DTT) plus NEM treatment annuls Zn(II) potentiation on rP2 × 4R. (**A**) quantification of the rP2 × 4R ATP evoked currents fold increase when co-applied with DTT 10–100 µM and representative traces of ATP evoked currents in absence and presence of 10 µM DTT (insert). (**B**) representative traces of ATP-evoked currents in absence (black) and presence of Zn(II) (Green) and after NEM + DTT treatment (red and blue) and (**C**) quantification of the fold increase of ATP-evoked currents by Zn(II). N = 4–6 DTT co-application, N = 6 for NEM + DTT treatment, * *p* < 0.05, ** *p* < 0.01, NS = non significative according to Mann–Whitney (A) or Kruskal–Wallis (C) test.

Preceding results strongly suggest that cysteine thiols are a requisite for the Zn(II)-induced modulation and that free thiols become available only upon oocyte incubation with Zn(II). To examine if the sole P2 × 4R reduction is sufficient to potentiate the ATP-evoked currents, we challenged oocytes with 10 µM ATP, followed 10 min later by a second ATP challenge co-applied with dithiothreitol (DTT); neither 10, 30, nor 100 µM DTT potentiated the ATP-evoked currents (Figure 4A). Larger DTT concentrations were toxic since the oocyte holding potential was lost, a likely acute toxicity indication.

We predicted that DTT should suppress the NEM-induced blockade of the Zn(II)-induced potentiation. To this aim, oocytes were challenged with ATP followed by the co-application of ATP plus Zn(II) to test the Zn(II) potentiation; 10 min later, 10 µM DTT was incubated with 300 µM NEM for 5 min. After 20 min washout, the ATP and ATP plus Zn(II) challenges were repeated (Figure 4B). As expected, Zn(II) potentiated 2.0 ± 0.2-fold the ATP-evoked currents (Figure 4C). After the combined DTT plus NEM treatment in the presence of Zn(II), the ATP-evoked currents were enhanced 3.9 ± 1.2-fold, similar to the Zn(II) plus NEM treatment. No potentiation of ATP-evoked currents by Zn(II) was observed (2.4 ± 0.7-fold for the first ATP application). This finding is similar to that attained after the NEM plus Zn(II) treatment (compare Figure 3C with Figure 2C), indicating that DTT and Zn(II) have similar macroscopic effects. Altogether, this set of data strongly supports that the P2 × 4R Zn(II)-induced ATP-evoked currents potentiation is a two-step kinetic process. First, head receptor domain disulfides make free thiols available followed by the metal four ligands coordination to cause the potentiation of the ATP-evoked currents.

### 2.4. The Zn(II)-Induced Modulation Is Lost in the C132A or C149A but Not the C126 and C159 Mutants; Effect of Double Cysteine Mutants

Based on the chemical protein modification protocol results, we next addressed which of the putative SS2 (C126 and C149) or SS3 (C132 and C159) cysteines are involved in the Zn(II)-induced modulation. To this aim, site-directed mutations of the SS2 and SS3 cysteines were replaced individually by alanine (A) or threonine (T) as in: C126A, C149A, C132A, or C159T mutants, in addition, double mutants C126A/C149A and C132A/C159A were also examined.

**Figure 5:**
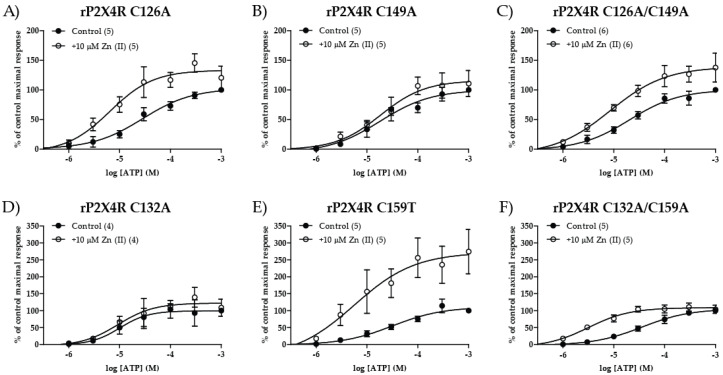
Cysteine residues 132 and 149 are relevant for the Zn(II)-induced positive allosteric modulation of rP2 × 4R. Single and double mutants for the SS2 (upper panel) and SS3 (down panel). (**A**,**B**) SS2 single mutants C126A and C149A concentration–response curves in absence (black) and presence (white) of 10 µM Zn(II). (**D**,**E**) SS3 single mutants C132A and C159T concentration–response curves in absence (black) and presence (white) of 10 µM Zn(II). (**C**,**F**) C126A/C149A and C132A/C159A double mutants concentration–response curves in absence (black) and presence (white) of 10 µM Zn(II). Number of experiments with each mutant are shown in parenthesis for concentration–response protocols. Symbols represent mean values, and bars S.E.M.

Regarding SS2 bond cysteines, as anticipated, the C126A mutant preserved Zn(II) modulation as evidenced by a 4.1-fold reduction in the EC_50_ from 37.9 ± 10.9 to 9.3 ± 3.0 µM (*p* = 0.032, *n* = 5, Figure 5A and Table 2) without a change in nH (*p* = 1, *n* = 5) or I_max_ (*p* = 0.069, *n* = 5, although *p* value was close to statistical significance). However, the C149A mutant (SS2 companion) was resistant to Zn(II) modulation since Zn(II) caused a modest 1.73-fold EC_50_ decrease (39.0 ± 16.7 v/s 22.5 ± 7.5 µM, control v/s + Zn(II), respectively, *p* = 0.55, *n* = 5; Figure 5B and Table 2). A similar finding had been observed by Li, et al., (2013) [31], but without ATP efficacy modification, revealing that this particular cysteine residue may play a critical role in the Zn(II) coordination. Compared to WT P2 × 4R, no difference was observed in the receptor parameters of these mutants, except that I_max_ doubled in the presence of the metal (Mann–Whitney test but *p* value was 0.055). Interestingly, the C126A/C149A double mutant, much to our surprise seemed to be modulated by Zn(II) since we observed a 2.26-fold decrease in the EC_50_ from 22.9 ± 6.7 to 10.1 ± 4.1 µM (*p* = 0.026, *n* = 6) without changes in nH or I_max_ (Figure 5C and Table 2).

Likewise, a similar detailed analysis was performed with SS3 cysteines. Consistent with previous publications [30,31] and as anticipated, the C132A mutant was resistant to Zn(II) modulation with no significant EC_50_ change in the presence of Zn(II) (*p* = 1, *n* = 4), nor nH (*p* = 0.69, *n* = 4) or I_max_ value (*p* = 0.89, *n* = 4) (Figure 5D and Table 2). Interestingly, the C159T mutant was Zn(II) sensitive as evidenced by a 5.32-fold EC_50_ reduction in the presence of the metal from 37.3 ± 8.8 to 7.0 ± 2.0 µM (*p* = 0.012, *n* = 5, Figure 5E and Table 2), without a significant modification of nH (*p* = 0.31, *n* = 5) but an I_max_ increase (4.1 ± 1.5 v/s 7.6 ± 2.3 µA, control v/s + Zn(II), respectively, *p* = 0.031, *n* = 5).

To further investigate this aspect, a series of cysteine double mutants were prepared which replaced SS3 cysteines by alanine but also by structurally related amino acids such as threonine or serine, the latter being structurally much closer to the cysteine, since the thiol was replaced by a primary OH. Much to our surprise, the C132A/C159A double mutant was even more sensitive to Zn(II)-modulation than the WT P2 × 4R as deduced from a 17.1-fold reduction in the EC_50_ by nucleotide co-application with Zn(II). The EC_50_ was reduced from 77.1 ± 24.1 to 4.5 ± 1.6 µM (*p* = 0.0079, *n* = 5; Figure 5F and Table 2) in the presence of the metal. No changes in the nH (*p* = 0.4, *n* = 5) or I_max_ (*p* = 1, *n* = 5) were observed. We next examined the threonine C132T/C159T double mutant which also resulted in Zn(II) sensitive based on a 5.5-fold EC_50_ reduction by the metal (27.4 ± 13.6 v/s 5.0 ± 0.9 µM, *p* = 0.0023, *n* = 7) with no significant changes in nH (1.5 ± 0.2 v/s 1.1 ± 0.2, *p* = 0.16, *n* = 7) or I_max_ (3.2 ± 0.7 v/s 4.3 ± 1.1 µA, *p* = 0.54, *n* = 7; Figure S3 left). Finally, the serine double mutant, C132S/C159S, which also proved sensitive to the metal as evidenced by a significant 3.41-fold reduction in its EC_50_ upon co-application with Zn(II) (20.5 ± 5.0 v/s 6.0 ± 2.2 µM, *p* = 0.032, *n* = 5) with no change in either nH (1.9 ± 0.5 v/s 1.4 ± 0.5, *p* = 0.55, *n* = 5) or I_max_ (4.1 ± 0.7 v/s 5.1 ± 1.1 µA, *p* = 0.42, *n* =5; Figure S3 right). Altogether, these three double mutants demonstrated Zn(II)-induced potentiation in the absence of SS3 thiols albeit to distinct extents, evidencing a similar profile as that observed with the SS2 double alanine mutant. None of these mutants showed significant differences on the P2 × 4R parameters compared to WT rP2 × 4R (Mann–Whitney test), evidencing that the SS2 or SS3 head receptor domain may be unfolded without significant changes in ATP potency.

### 2.5. E160 and D170 Relevance for the Zn(II) Modulation; Poisson–Boltzmann P2 × 4R Electrostatic Potential Calculations 

**Figure 6:**
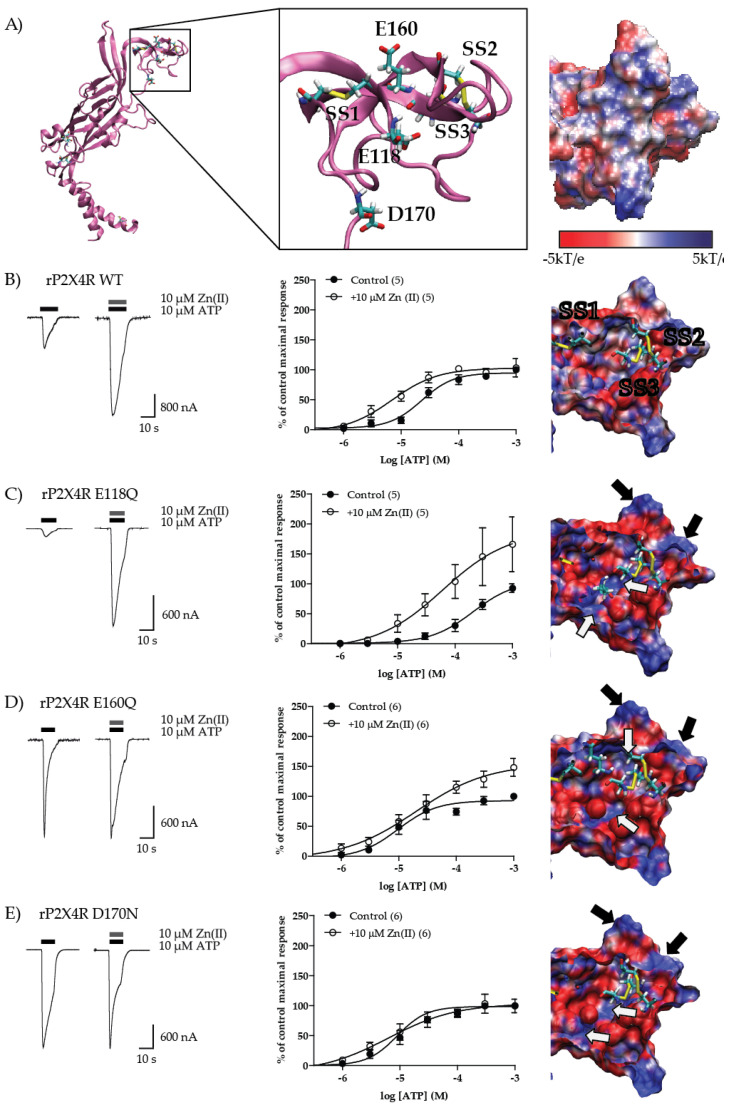
Electrophysiological recordings and electrostatic potential of the rP2 × 4R and E118Q, E160Q, and D170N mutants. (**A**) Representation of a rP2 × 4R monomer with SS1–5 (left) and enclosure of the head domain showing SS1-3 and acid residues (midle), with the external surface electrostatic potential (right). (**B**–**E**) Left panel, representative traces of the 10 µM ATP evoked currents in the absence and in the presence of 10 µM Zn(II), the middle panel shows the ATP concentration–response curves in absence (black) and presence (white) of 10 µM Zn(II); the right panel shows the electrostatic potential in head domain of each receptor mutant (please note the SS1, SS2 and SS3 as reference) for the WT receptor and E118Q, E160Q, and D170N P2 × 4R mutants, respectively. The arrows in the right column show the most obvious changes on the electrostatic potential, black arrows for the surface, and white arrows for protein residues inside the model. Symbols on the graphs represent mean values, bars S.E.M. In parenthesis, is a number of replicas of each ATP curve. Note that D170 is not shown in the E right panel because it is located in the linker region immediately beneath the head domain (see the magnification in (**A**)). All electrostatic calculations were made in the full trimeric P2 × 4R and mutants; we show the head domain of a monomer as a simplified model.

The finding that only non-charged thiol alkylators annulled the P2 × 4R Zn(II)-induced potentiation suggested that the access to the head receptor domain is dependent on negatively charged P2 × 4R residues. We inferred a negatively charged microenvironment in the immediate SS2/SS3 neighborhood necessary for Zn(II) access to this site. To ascertain the verisimilitude of this hypothesis, we modeled the P2 × 4R based on the *Danio rerio* 3I5D structure [27] and analyzed the electrostatic potential of the head receptor domain with the adaptative Poisson–Boltzmann solver (APBS). Results indicate that the SS1, SS2, and SS3 surroundings have a negative electrostatic potential (Figure S5); in contrast, the orthosteric ATP site, showed a positive electrostatic potential, compatible with the triphosphate ATP moiety. We favored the use of the current spatial structural model because it lacks the SS4 and SS5 compared to other tridimensional resolutions [37]. To identify whether acid residues located near the P2 × 4R head domain are relevant to determine a negative electrostatic potential to attract a divalent metal, we examined the influence of acid residues E118 and E160 in the SS2/SS3 vicinity (Figure 6A); the role of these acids had not been evaluated before. As a strategy to cause a minimal volume perturbation in the head receptor domain, in-silico mutations of these acid residues were replaced by the respective amides (i.e., E by Q and D by N) as in mutants E118Q, E160Q. In addition, D170N was preferred over D170A since the latter did not abolish the Zn(II)-induced ATP-evoked currents potentiation [38]. The electrostatic potential of the WT receptor and mutants is visually shown in a color-graded scale from −5 kT/e (red, negative potential) to 5kT/e (blue, positive potential).

To discover whether E118 and E160 are critical for the Zn(II) modulation, ATP concentration–response protocols were performed in the absence and presence of 10 µM Zn(II). Mutant E118Q, was 21.9-fold less potent than the WT (EC_50_: 633 ± 228 v/s 27.5 ± 10.6 µM, respectively, *p* = 0.016, *n* = 5, Table 2), revealing that, while E118 is close to SS2 or SS3, its mutation by a noncharged residue significantly diminished ATP potency or altered P2 × 4R channel gating. Notwithstanding, the co-application of 10 µM Zn(II) shifted leftwards 13.6-fold the ATP concentration–response curve from 633 ± 228 to 55.2 ± 23.0 µM (*p* = 0.016, *n* = 5; Figure 6C and Table 2) without altering nH (*p* = 0.69, *n* = 5) or I_max_ (*p* = 0.84, *n* = 5, Table 2), suggesting that this residue likely plays a more relevant role in the agonist’s affinity rather than in metal modulation. In matched, paired control oocytes, co-application of ATP plus 10 µM Zn(II) potentiated the ATP-gated currents (Figure 6B), shifting leftwards, in a parallel manner the EC_50_ 3.45-fold from 27.5 ± 10.6 to 8.3 ± 3.0 µM (*p* = 0.031, *n* = 5; Figure 6B and Table 2) without modifying the I_max_ (*p* = 0.5, *n* = 5) or nH values (*p* = 0.5, *n* = 5). Regarding the electrostatic potential, as expected, E118Q shifted the charge density to more positive values compared to the WT P2 × 4R (compare colored analysis in Figure 6B,C), suggesting that modifying the charge density of this residue is not necessarily linked to the Zn(II) modulation but to the markedly reduced agonist potency. In contrast, both E160Q and D170N conserved ATP potency; no changes were observed neither in EC_50_s, nH, or I_max_ compared to WT (Table 2, Mann–Whitney test). Notwithstanding, E160Q mutant proved resistant to Zn(II) modulation as evidenced by similar EC_50_ values with or without the metal (*p* = 0.48, *n* = 6; Figure 6D and Table 2); nH (*p* = 0.39, *n* = 6) or I_max_ (*p* = 0.39, *n* = 6) values were not modified. E160Q showed an electrostatic potential shifted to more positive values (Compare Figure 6B with Figure 6D, right column), indicating that in this mutant, the SS2/SS3 vicinity is less favorable for Zn(II) attraction, a finding fully compatible with the loss of metal modulation in E160Q. Interestingly, D170N shifted the electrostatic potential to more positive values, as anticipated (compare the colored potential between Figure 6B,E) and was resistant to Zn(II) modulation. The EC_50_ ratio in the metal presence was only 1.5 (19.3 ± 10.5 v/s 12.8 ± 8.1 µM *p* = 0.31, *n* = 6); with unaltered nH and Imax (Figure 6E and Table 2). Altogether, these findings support the notion that residues E160 and D170 participate in the metal modulation mechanism, providing a negative charge density microenvironment required for metal attraction to the head receptor domain.

## 3. Discussion

A precise consensus molecular mechanism for the Zn(II)-mediated P2 × 4R allosteric modulation is still lacking; this research adds novel information regarding the role of sulfhydryl groups in the head receptor domain sensitive to either NEM or IA alkylation. Chemical protein modifications occurred only when P2 × 4R was co-incubated with these reagents plus Zn(II); covalent chemical modification was verified as the long-lasting annulment of the Zn(II)-induced modulation. In contrast, DTT abrogated NEM effects, confirming the relevance of protein thiols in the metal modulation. As anticipated from the working hypothesis, protein alkylators did not modify the Zn(II)-induced allosteric modulation of P2 × 4R when reagents were applied in the absence of Zn(II), discarding nonspecific alkylation’s of other protein side groups or environmental membrane proteins required for receptor functionality. Moreover, parallel studies used P2 × 2Rs as a further control to eliminate the possibility of nonselective chemical reactions, showing that the Zn(II)-induced potentiation was conserved even after NEM treatment because the effect of Zn(II) in the P2 × 2R is due to coordination with imidazole’s from critical histidine residues [35,36] which apparently are not NEM-sensitive. Chemical protein modification was further supported by site-directed mutagenesis of head domain cysteines, clearly establishing the role of C132 and C149 in the possible allosteric mechanism, discarding the participation of C126 or C159. In addition, carboxylic acid residues from E160, and to a minor extent D170, are also required for the Zn(II) positive allosteric modulation discarding the participation of E118. Regarding the mutation of head domain cysteines, although previous studies indicated that individual mutations of the P2 × 4R SS2 and SS3 bonds are relevant for ATP efficacy [39], current findings, based on full concentration–response studies, only support the role of C149 in ATP efficacy.

An interesting observation that cannot be overlooked because it is reported in the literature and repeatedly observed in the present results, refers to the finding that the single mutation of either C132 or C149 for alanine, suffices to obliterate the Zn(II)-mediated allosteric interaction. It should be evident by the mutants, as well as the P2 × 4R modelling, that the thiols susceptible of Zn (II) coordination are crossed; while C149 belongs to the SS2, C132 is derived from the SS3 bond. Based on this finding, we deduce the need that both SS2 and SS3 must be somehow reduced to allow cross interactions between C132 and C149. We hypothesize that it is likely that this pair of thiols match perfectly the distance and contour required for Zn(II) coordination. Consequently, the loss of either C132 or C149 determines the loss of the allosteric effect. In contrast, we were puzzled by the repeated observation that the SS2 or the SS3 double mutants such as C126A/C149A or C132A/159A, or the SS3 mutants C132T/C159T or C132S/C159S, all sustained the Zn(II) modulation, allowing us to suppose that a significant head receptor domain conformational rearrangement must occur in the absence of either SS2 or SS3. Among other plausible explanations, we hypothesize that perhaps the lack of one of the disulfides plus its artificial replacement by either alanine, serine, or threonine causes a major perturbation of the head receptor domain microenvironment that, unexpectedly, makes possible that Zn(II) coordinates with the two thiols derived either from the SS2 or SS3, respectively, allowing the allosteric conformational change subsequent to the Zn(II) coordination. It is obvious that the quadruple mutant, if viable, should resolve this paradigm; however, we dissented on its synthesis, conscious that this mutant will cause a very major head receptor domain distortion. We visualize based on this interpretation, that in the presence of Zn(II), not all four thiols may be in fact be alkylated by either NEM or IA alkylation, due perhaps to the steric hindrance caused by one or two NEM molecules covalently attached in the limited head receptor domain area.

Therefore, we propose that in the presence of Zn(II), a fraction of the head receptor domain disulfide bonds must be in the reduced state providing free thiols prone to reaction with non-charged alkylators. Both NEM and IA conform to this interpretation, while IAc or DTNB did not annul the allosteric role of Zn(II). We deduce that since the latter two alkylators are negatively charged, they do not readily have access to the accessible thiols as the non-charged alkylators NEM or IA do. In the absence of Zn(II), treatment with 2-(trimethylammonium)ethyl methanethiosulfonate (MTSET), a positively charged reagent, was reported to diminish the Zn(II) potentiation [30], indicating the relevance of the charge in the access to the alkylators to their site of action. Furthermore, MTSET did not completely abolish metal potentiation, confirming that Zn(II) is needed in the treatment to annul the positive allosteric modulation. The finding that treatment with IA, but not NEM, in presence of Zn(II) abolished the Zn(II)-mediated allosteric P2 × 2R modulation may be interpreted as proof of the extended IA reactivity to imidazoles, since IA and IAc are known to react with other nucleophilic chains [40]. The experiment with DTT reveals that the sole reduction of P2 × 4R disulfide bonds is not sufficient to potentiate the ATP-gated currents [41] and the treatment with NEM plus DTT mimics the effect of NEM plus Zn(II) treatment. The latter observation supports our notion that the allosteric modulation mechanism is grounded on two sequential chemical steps: (i) availability of thiols in the head receptor domain followed by (ii) metal coordination with these ligands to elicit P2 × 4R conformational changes compatible with the increased ATP potency caused by the allosteric modulation. The NEM logP (an estimate of octanol:H_2_O partition) is 0.02 according to ALOGSP2.1 (Virtual Computational Chemistry Laboratory) [42,43] and, therefore, the likelihood of NEM permeating intracellularly following a short incubation is rather limited, and we cannot definitely rule out intracellular alkylations.

An issue that needs to be further addressed questions how the head domain SS2/SS3 provides the free SH groups necessary as ligands for metal coordination. Our current interpretation is centered on the probability that the SS2/SS3 bonds might be in continual equilibrium between the oxidized/reduced species depending on prevailing microenvironmental conditions. The presence of Zn(II) somehow favors the generation of free thiols that will act as Zn(II) ligands. Several scenarios deserve discussion: (i) Zn(II) binding to an as yet undetermined anionic site in the head domain vicinity changes the P2 × 4R conformation, introducing tension to the SS bonds causing a subsequent disulfide bridge cleavage; (ii) this metal effect may be aided by an endogenous, extracellular, reducing agent to favor SS2/SS3 reduction; or (iii) a fraction of these disulfide bonds spontaneously oscillates in a continual and dynamic redox equilibrium. Disulfide bond reduction may be assisted by extracellular glutathione, a tripeptide with antioxidant functions that is localized both intracellularly and extracellularly [44]. As a second option, we deem it to be possible that a fraction of the P2 × 4R head domain disulfide bonds may be in a redox balance between free SH and its oxidized form as demonstrated for psoriasin (S100A7), which is released from epithelial cells in two forms, one oxidized and another reduced [45]. We are aware that Zn(II) cannot reduce disulfides (as it does for instance metallic zinc [46]) but our data strongly supports that somehow the alkylation process depends on Zn(II). Obviously, this issue should be resolved by determining the eventual alkylation of head receptor domain cysteines with labeled NEM or IA in the presence of Zn(II) followed by P2 × 4R fingerprinting to determine precisely the alkylated residues. In addition, our experimental design does not allow one to titrate the ratio of NEM or IA SH alkylation, nor the free thiol localizations.

We have not overestimated alternative explanations to our proposal based on thiols as main Zn(II) ligands in the head receptor domain. We are aware from crystallography and electrophysiological data that Glu95 coordinates Zn(II) acting at a distinct allosteric site which involves inter subunit coordination with adjacent Glu95 residues [38]. We deem it theoretically possible that Zn(II) might bind to more than one site accounting for its strong metal allosteric modulation. The possible prevalence of one site over the other must depend on the relative metal affinity and metal availability at either site. We are keen to address this issue, which might reveal unexpected intricacies of metal protein interaction mechanisms. The Glu95 site has not been properly characterized on P2 × 4R and awaits further supporting experimental evidence. Notwithstanding, the Glu95 site cannot account for the present observations on P2 × 4R alkylation by NEM or IA co-incubated with the Zn(II), or the loss of the Zn(II)-mediated allosteric effect in C132A or C149A mutants abolishes Zn(II) modulation, favoring the suggestion that perhaps, Zn(II) acts simultaneously at two sites to account for its strong positive allosteric modulation.

In addition to the putative role of head domain disulfides, we also examined whether carboxylic residues in the immediate neighborhood might also participate or contribute in the Zn(II) coordination sphere. Based on the receptor bioinformatic modelling, we became increasingly conscious of the close distance of the free carboxylic acid E160 to the head domain thiols. Therefore, we examined the contribution of neighboring acid residues in Zn(II)-mediated allosteric modulation. With regard to the role of D170, Kasuya et al., (2016) showed that the D170A mutant was viable and reported a 2-fold potentiation of the 3 µM ATP-gated currents, but no full ATP concentration–response studies, nor Zn(II) modulation protocols were performed [38]. Moreover, the role of this residue was established for the PPADS sensitivity on P2 × R [47]. The present protocols showed that 10 µM Zn(II) did not displace significantly the ATP concentration–response curve, an indication that this mutant lost the Zn(II)-mediated allosteric response. In addition, receptor modelling, based on Latapiat et al., (2017) [32], allowed visualizing the spatial localization of this residue, the putative influence of E160 and D170 as Zn(II) ligands in the vicinity of SS2/SS3 bonds. Considering the head P2 × 4R domain, the D170 carboxylic group is at 18.7 Å from the SS3 in the apo and 18.8 Å in the holo P2 × 4R configuration (distances measured in the full trimeric models), a distance far enough from the C132 to invalidate its role in the metal coordination complex (based on the notion that the C132 sulfhydryl is a putative coordination ligand). A likely interpretation of the present data supports that D170 is determinant for the P2 × 4R head domain electrostatic potential, rather than as a metal coordination ligand. Notwithstanding, the E160Q mutant is, likewise, resistant to Zn(II) modulation, as evidenced by its non-significant EC_50_ shift by 10 µM Zn(II). In our P2 × 4R model, the E160 free carboxyl group is at 9.53 Å from C132 in the apo versus 7.22 Å in the holo rP2 × 4R, a distance much closer than D170, but still far from C132 allowing us to discard this carboxyl group as a putative Zn(II) ligand. It is evident from the combination of Poisson–Boltzmann equation solving and electrophysiology, that not every acid residue in the head receptor domain contributes equally to the charge density and/or Zn(II) modulation in the SS2/SS3 vicinity. This finding supports a role of E160 and D170 in the electrostatic potential determination. In addition, the E118Q in-silico mutation showed a charge density modification on the rP2 × 4R head domain, but this mutant is clearly sensitive to Zn(II) modulation, and, therefore, can be discarded as a putative coordination ligand. The observed increase in ATP affinity by the mutant may be due to a modification in the electrostatic potential of the orthosteric site.

Moreover, and based on the notion that the metal most common coordination geometry is tetrahedral [48], we reason that C149 which is spatially only at 4.62 Å from C132, may be likely a metal ligand candidate, together with an undetermined additional residue plus one or two water molecules. This new proposal highlights the relevance of the head receptor domain disulfide residues in the Zn(II)-mediated allosteric modulation. In sum, present findings allow us to propose in a biophysical background the key role of amino acid residues in the P2 × 4 head receptor domain such as C132 and C149, plus E160 and D170 in the Zn (II) allosteric modulation. This novel molecular proposal suggests a Zn(II) coordination model based on two sequential steps: (i) chemical attraction to a fraction of “free thiols” from SS2/SS3 followed by (ii) Zn(II) coordination to cause subsequent P2 × 4R conformational changes compatible with the allosteric modulation. In addition, the proposal sets new perspectives for the modulator role of Zn(II) in proteins, relevant for other biophysics mechanisms supporting metal applications.

## 4. Materials and Methods

### 4.1. Animals and Oocyte Harvesting

Adult *Xenopus laevis* frogs housed at a maximum density of 3 animals per 24 L of chloride-free water with controlled room temperature (20 ± 1 °C). Animals where fed with low fat meat twice weekly. Surgical extraction of an ovary lobule segment was performed in fully anesthetized animals under 0.15% tricaine anesthesia. Each animal was operated on a maximum of four times with a recovery period of at least 5 weeks between surgeries. After the surgical procedure, animals were monitored for 2–3 h on an independent pool, prior to placed back to routine housing. All animal procedures were approved by the Universidad de Santiago ethics committee (resolution 484, September 2016), in agreement with NIH guidelines.

After the ovary extraction, stage V-VI oocytes where manually defolliculated followed by a 20 min incubation with 1 mg/mL type 1A collagenase. 5 ng of a plasmid containing the coding sequence for the rat P2 × 4 receptor (rP2 × 4R), E118Q, E160Q, D170N, C126A, C132A, C149A, C159T, C126A/C149A, C132A/C159A, C132T/C159T, and C132S/C159S mutants or rat P2 × 2 receptor (rP2 × 2R) where microinjected intranuclearly on each oocyte using a manual microinjection pipette from Drummond Scientific Company (Broomall, PA, USA). Microinjected oocytes where held on Barth’s buffer (88 mM NaCl, 1 mM KCl, 2.4 mM NaHCO_3_, 10 mM HEPES, 0.82 mM MgSO_4_, 0.33 mM Ca(NO_3_)_2_, 0.91 mM CaCl_2_, pH 7.4) supplemented with 2 mM sodium pyruvate and 10 UI/L penicillin and 10 mg/L streptomycin at 16 °C until the electrophysiological recordings.

### 4.2. Chemicals and Plasmids

Sodium chloride (NaCl), potassium chloride (KCl), bicarbonate (NaHCO_3_), magnesium sulfate (MgSO_4_), calcium chloride (CaCl_2_), and calcium nitrate (Ca(NO_3_)_2_) where purchased from Merck (Darmstadt, Germany). HEPES, zinc chloride (ZnCl_2_), adenosine triphosphate as the sodium salt, sodium pyruvate, collagenase 1A, dithiothreitol (DTT), and N-ethylmaleimide (NEM) were purchased from Sigma-Aldrich (St Louis, MO, USA). 5, 5’-dithiobis-(2-nitrobenzoic) acid (DTNB) was purchased from Abcam (Cambridge, UK). Iodoacetamide (IA) was provided by Prof. J. Eyzaguirre and Iodoacetate (IAc) by Prof. E. Silva. Penicillin/Streptomycin was purchased from Thermo Fisher Scientific (Waltham, MA, USA). rP2 × 4 receptor coding sequence was synthetized and cloned on a pcDNA3 plasmid (GenScript, Piscataway, NJ, USA). Site-directed mutagenesis E118Q, E160Q, D170N, C126A, C149A, C126A/C149A, C132A/C159A, and C132S/C159S were performed by GenScript (Piscataway, NJ, USA); the fidelity of the clone was checked in each case by the respective sequencing. The C132A mutant was previously synthetized by our group and is the same published on previous reports [30,32] while the C159T and C132T/C159T where kindly provided by Dr. Hana Zemková [39]. The rP2 × 2R was also previously synthesized by our group [49].

### 4.3. Two Electrode Voltage Clamp Recordings

Stages V or VI oocytes were collected to express WT rP2 × 4R or mutants; a two-electrode voltage clamp was used to examine Zn(II) allosteric modulation as reported [30]. After at least 36 h incubation at room temperature in Barth’s buffer (in mM: 88 NaCl, 1 KCl, 2.4 NaHCO_3_, 10 HEPES, 0.82 MgSO_4_, 0.33 Ca(NO_3_)_2_, and 0.91 CaCl_2_, pH 7.4) supplemented with 10 IU/L penicillin/10 mg streptomycin and 2 mM pyruvate, oocytes were putted individually in a 100 µL register chamber with a constant Barth’s perfusion of 2 mL/min and clamped at −70 mV using an OC-725C clamper (Warner Instruments); the signal was digitalized at 1 KHz with a MiniDigi 1B (Axon CNS, Molecular Devices, San Jose, CA, USA). Pipet electrodes (Kwik-fill, World Precision Instruments, Sarasota, FL, USA) were filled with 3M KCl; resistance ranged from 0.8 to 2 MΩ.

#### 4.3.1. Allosteric Modulation Protocols

Allosteric modulation protocols were performed following co-application of 10 µM Zn(II) plus varying ATP concentrations ranging from 0.3 µM to 1 mM in rP2 × 4R and E118Q, E160Q, D170N, C126A, C132A, C149A, C159T, C126A/C149A, C132A/C159A, C132T/C159T, and C132S/C159S mutants. ATP or ATP plus Zn(II) co-applications (during 10 s each) were delivered after at least 10 min washout. Mutants replacing cysteines by alanine, threonine, or serine eliminated disulfide bonds and/or Zn(II) ligand; mutants replaced acid residues by neutral side groups such as glutamine (Q) or asparagine (N), eliminating the negative charge rather than influencing volume modifications. GraphPad Prism 5 (GraphPad Software Inc., San Diego, CA, USA) assisted Hill equation fitting (Y = Bottom + (Top − Bottom)/(1 + 10^((LogEC_50_ − X) × HillSlope))) to calculate EC_50_, nH and I_max_ values for each concentration–response protocol. The same oocyte was routinely used to derive a complete concentration–response curve in the absence and later in presence of 10 µM Zn(II). Oocytes that did not endure this double protocol were discarded (30/113). Oocytes batches from at least two separate frogs were used to obtain pharmacological parameters for WT rP2 × 4R or mutants.

#### 4.3.2. Thiols Alkylation Protocols

To evaluate the effect of cysteine alkylation on the Zn(II)-induced modulation, we performed a full concentration response curve on oocytes expressing the rP2 × 4R or rP2 × 2Rs. For rP2 × 4Rs, after the first full ATP concentration curve was accomplished, the same oocyte was co-incubated with vehicle (Barth’s buffer), or 300 µM NEM plus vehicle or 300 µM NEM plus 10 µM Zn(II). In the case of P2 × 2Rs, 300 µM NEM was co-incubated with either vehicle or 30 µM Zn(II) for 5 min and extensively washout with Barth’s buffer for 20 min. After each treatment, the same oocytes where challenged with another concentration–response curve in the (i) absence or (ii) the presence of Zn(II) (10 µM for rP2 × 4R and 30 µM for rP2 × 2R) or (iii) both. In the case of iodoacetamide (IA), the same protocol used for NEM was followed, including co-incubation with 300 µM IA. Likewise, the effect of 300 µM DTNB was also tested on rP2 × 4R expressing oocytes following the same protocol in the presence or absence of 10 µM Zn(II).

The effect of cysteine alkylating agents on Zn(II) potentiation was tested on rP2 × 2R and rP2 × 2R expressing oocytes, for that purpose we challenge the oocytes with 10 µM ATP and after 10 min of washout we tested 10 µM ATP + 10 µM Zn(II) for metal potentiation. After 10 min of washout we applied 300 µM NEM, 300 µM IA, 300 µM IAc, or 300 µM DTNB for 5 min in absence or presence of Zn(II) (10 µM for rP2 × 4R and 30 µM for rP2 × 2R). After an extensive 20 min washout with Bart’s buffer we tested again the ATP and ATP + Zn(II) challenges separated by 10 min.

To evaluate if ATP-evoked currents are potentiated by disulfide bonds reduction we challenged rP2 × 4R expressing oocytes with 10 µM ATP, washed out for 10 min and make a second challenge with 10 µM ATP in presence of 10 µM 30 µM or 100 µM DTT, larger concentrations where toxic for oocytes. In order to test if ATP-evoked currents are potentiated by Zn(II) after DTT treatment we challenge the oocytes with 10 µM ATP and after 10 min of washout we tested 10 µM ATP + 10 µM Zn(II) for metal potentiation. After 10 min of washout we applied 300 µM NEM + 10 µM DTT. After an extensive 20 min washout with Barth’s buffer we tested again the ATP and ATP + Zn(II) challenges separated by 10 min.

### 4.4. Statistics

All data set distributions were found to be not-normal according to the Shapiro–Wilk test. EC_50_, nH, I_max_, EC_50_ ratio, and Zn(II) potentiation where compared between the same sample (i.e., with v/s without Zn(II)) and against the WT rP2 × 4 with Mann–Whitney test. For multiple comparisons we use Kruskal–Wallis test followed by Dunn’s post-test. Data sample number and *p* value are shown in the text and/or figures.

### 4.5. Molecular Modeling and Electrostatic Potential Calculations

A molecular rP2 × 4R model was constructed based on the crystallized *Danio rerio* P2 × 4R structure (PDB: 3I5D) [27] and the P51577-1 rat sequence using the Modeller 9.15 program [50]. The utilized structure was chosen by Protein Blast alignment (blastp, NCBI, Bethesda, MD, USA). The zebra fish ortholog has 62% identity with the rat sequence; the N and C-terminus were split from the model (amino acids 1–30 and 138–388, respectively). Trimeric construction and refinement were assisted with GalaxyGemini program from the GalaxyWeb server [51]. Model quality was ascertained with ProSA-web [52] and RAMPAGE [53] obtaining a Z value of −6,13, with 78.3% of residues in a favorable region, 16.4% of the residues in an allowed region and 5.3% in an outlier region. In-silico mutants were performed with the MUTATOR program of VMD 1.9.1 (Theoretical and Computational Biophysics Group). After each mutation, the models where prepared again for following the minimization steps. Protonation state of titratable residues been taken into account when preparing each model. In total, 50,000 energy minimizations steps were carried out for the WT receptor and mutants as described [32]. The Adaptive Poisson–Boltzmann Solver (APBS 1.4.2.1) [54,55] assisted rP2 × 4R WT and mutant electrostatic potential calculations; a color graded scale from −5 to 5 kT/e was used. In addition, the model was also used to measure distances between carboxyl group of acid residues and C sulfhydryl groups in the apo (close) and holo (open) rP2 × 4R states, for that purpose an open rP2 × 4R model was constructed based on the 4WD1 structure [28] following the previous steps. Z value was −6.21 with 97.8 residues in a favorable region, 1.8% in an allowed region and 0.3% in an outlier region. Visualization and distances measurement were carried out using the VMD 1.9.1 program (Theoretical and Computational Biophysics Group). All distances measurements and electrostatic potential calculations were performed using the full trimeric models.

## Figures and Tables

**Table 1:** Pharmacological characterization of the wild type rP2 × 4R and rP2 × 2 before and after NEM or NEM + Zn(II) treatments.

		NEM Treatment + Vehicle, ±SEM			NEM Treatment + Zn(II), ±SEM		
		**EC_50_ (µM)**	**nH**	**I_max_ (µA)**	**EC_50_ (µM)**	**nH**	**I_max_ (µA)**
**rP2 × 4R**	**Control before treatment**	23.0 ± 8.3 (9)	1.9 ± 0.4	3.5 ± 0.7	24.5 ± 9.1 (6)	1.5 ± 0.4	6.6 ± 1.1
	**Control**	23.8 ± 7.0 (6)	2.8 ± 0.4	3.7 ± 1.2	44.9 ± 17.7 (6)	1.9 ± 0.4	8.9 ± 1.4
	**+Zn(II)**	3.0 ± 0.9 *^,‡‡^ (5)	2.0 ± 0.6	9.0 ± 0.7 *^,‡^	24.0 ± 8.8 (6)	1.7 ± 0.5	9.3 ± 2.2
**rP2 × 2R**	**Control before treatment**	67.7 ± 13.3 (5)	1.9 ± 0.5	29.5 ± 2.6	39.9 ± 8.3 (7)	1.8 ± 0.3	26.7 ± 3.5
	**Control**	35.0 ± 9.2 (4)	1.9 ± 0.3	30.8 ± 5.5	33.0 ± 7.4 (5)	1.9 ± 0.3	34.8 ± 4.5
	**+Zn(II)**	7.1 ± 1.9 *^,‡^ (3)	1.8 ± 0.4	30.6 ± 3.4	8.0 ± 1.5 **^,‡^ (6)	1.7 ± 0.1	33.1 ± 2.7

* *p* < 0.05, ** *p* < 0.01 when compared to its own control, ^‡^ *p* < 0.05, ^‡‡^ *p* < 0.01 when compared with control pre-treatment; according to Kruskal–Wallis test. *N* is indicated in parenthesis. Please note that this results are grouped by treatment, Control = ATP curve after treatment, Control before treatment = first ATP curve, + Zn(II) = ATP curve in presence of Zn(II) post treatment.

**Table 2:** Pharmacological characterization of the wild type rP2 × 4R and mutants before and after Zn(II) co-incubation with ATP.

	Control, ±SEM			+10 µM Zn(II), ±SEM		
RECEPTOR	**EC_50_ (µM)**	**nH**	**I_max_ (µA)**	**EC_50_ (µM)**	**nH**	**I_max_ (µA)**
P2 × 4 WT (5)	27.5 ± 10.6	1.3 ± 0.2	4.8 ± 0.9	8.3 ± 3.1 *	1.3 ± 0.2	4.8 ± 1.8
E118Q (5)	624 ± 232 ^Ŧ^	1.1 ± 0.3	4.1 ± 1.1	54.3 ± 23.1 *	0.9 ± 0.2	5.6 ± 2.0
E160Q (6)	23.8 ± 10.1	1.7 ± 0.5	4.0 ± 0.9	25.9 ± 7.1	1.5 ± 0.6	6.6 ± 1.8
D170N (6)	19.3 ± 10.5	1.7 ± 0.3	5.7 ± 0.8	12.8 ± 8.1	1.1 ± 0.2	5.6 ± 0.7
C126A (5)	37.9 ± 10.9	1.5 ± 0.6	7.9 ± 2.7	9.3 ± 3.0 *	0.9 ± 0.2	11.7 ± 4.8
C149A (5)	39.0 ± 16.7	1.8 ± 0.4	8.4 ± 1.2	22.5 ± 7.5	1.3 ± 0.3	10.7 ± 3.5
C132A (4)	10.2 ± 3.8	2.5 ± 1.0	6.5 ± 1.1	19.9 ± 13.4	2.5 ± 0.6	7.0 ± 1.9
C159T (5)	37.3 ± 8.8	2.0 ± 0.7	4.1 ± 1.5	7.0 ± 2.0 *	0.9 ± 0.3	7.6 ± 2.3 *
C126A/C149A (6)	22.9 ± 6.7	1.4 ± 0.3	5.3 ± 1.2	10.1 ± 4.1 *	0.9 ± 0.1	6.8 ± 1.3
C132A/C159A (5)	77.1 ± 24.1	0.9 ± 0.2	7.1 ± 2.1	4.5 ± 1.6 **	1.3 ± 0.3	7.4 ± 2.4

* *p* < 0.05, ** *p* < 0.01, compared to its own control; ^Ŧ^ *p* < 0.05, respect to WT according to Mann–Witney test. N are indicated in parenthesis.

## Supplementary Materials

Supplementary materials can be found at https://www.mdpi.com/1422-0067/21/18/6940/s1.
