# Supplementary material for: New Insights of the Zn(II)-Induced P2 × 4R Positive Allosteric Modulation: Role of Head Receptor Domain SS2/SS3, E160 and D170
Source: Int J Mol Sci. 2020 Sep 22;21(18):6940. doi: 10.3390/ijms21186940 (PMC7555825; doi:10.3390/ijms21186940)
Supplement: Supplementary file 1 [file ijms-21-06940-s001.pdf]

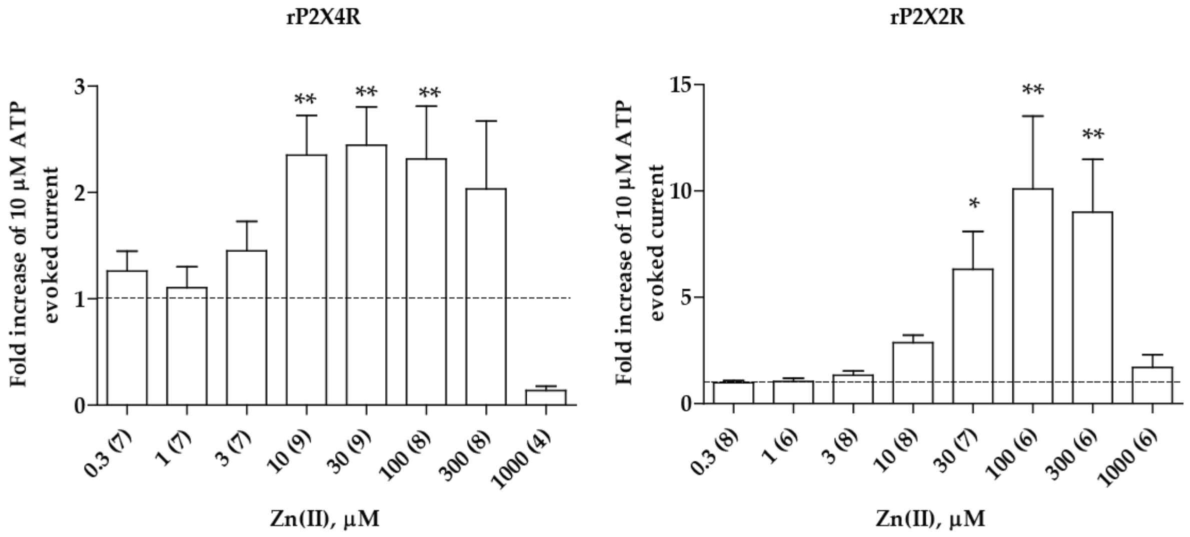

Figure S1. Zn(II) potentiates ATP-evoked currents on rP2X4R and rP2X2R. Quantification of the fold increase of ATP evoked currents by co-application of Zn(II) in rP2X4R (**left**) and rP2X2R (**right**) expressing oocytes. * $p < 0.05$ and ** $p < 0.01$ according with Kruskal-Wallis test.

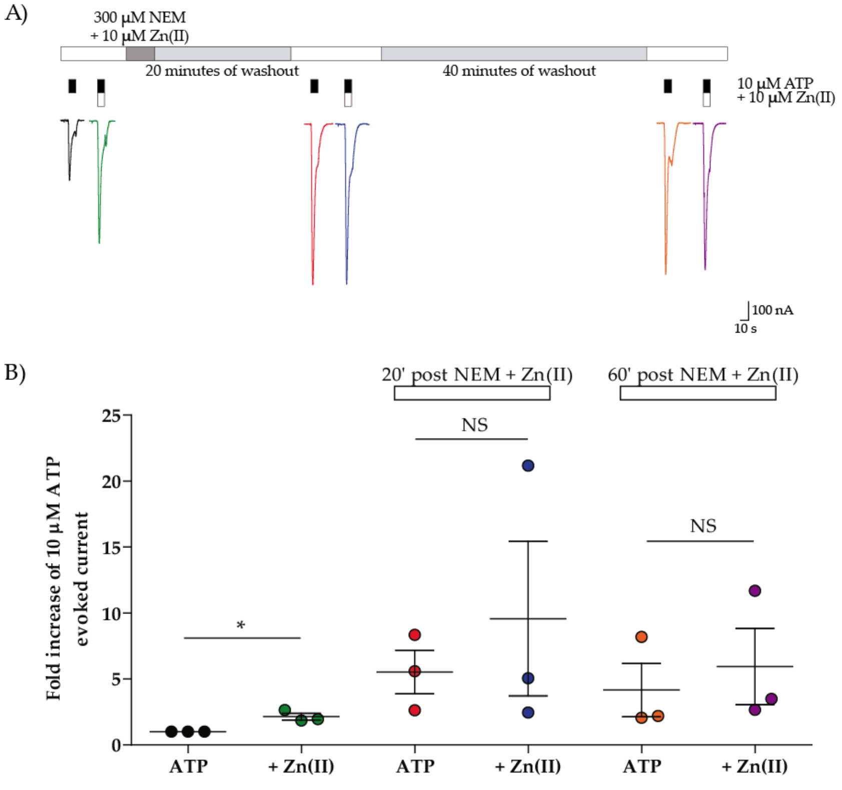

Figure S2. NEM + Zn(II) treatment abolish Zn(II) potentiation in rP2X4R after 1 h 30 m. (**A**) representative traces of ATP-evoked currents in absence (black) and presence of Zn(II) (green) before NEM + Zn(II) treatment, immediately after NEM + Zn(II) treatment (red and blue) and 1 h after the treatment (orange and purple). (**B**) quantification of the fold increase of ATP-evoked currents by Zn(II) before, immediately after and 1 h after NEM + Zn(II) treatment. * $p < 0.05$, NS = non significative according to Kruskal-Wallis test.

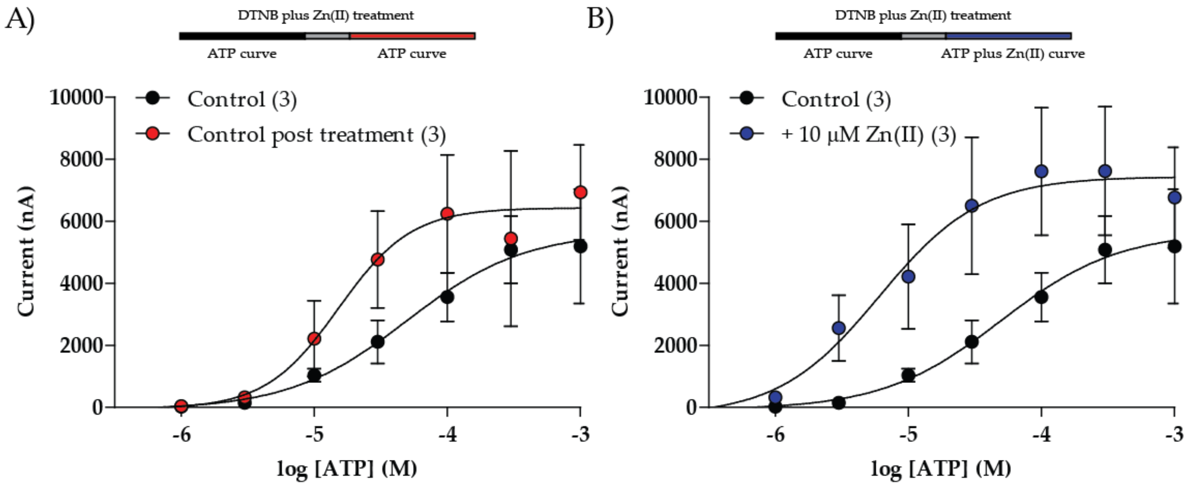

Figure S3. DTNB + Zn(II) treatment did not abolished the Zn(II) modulation in rP2X4R. (**A**) concentration-response curves before (black circles) and after (red circles) DTNB + Zn(II) treatment. (**B**) comparison of concentration-response curves in presence of Zn(II) after DTNB + Zn(II) treatment (blue circles) with the control pre-treatment (black circles). Treatment consist on 300 μM DTNB + 10 μM Zn(II) for 5 min followed by 20 min of washout.

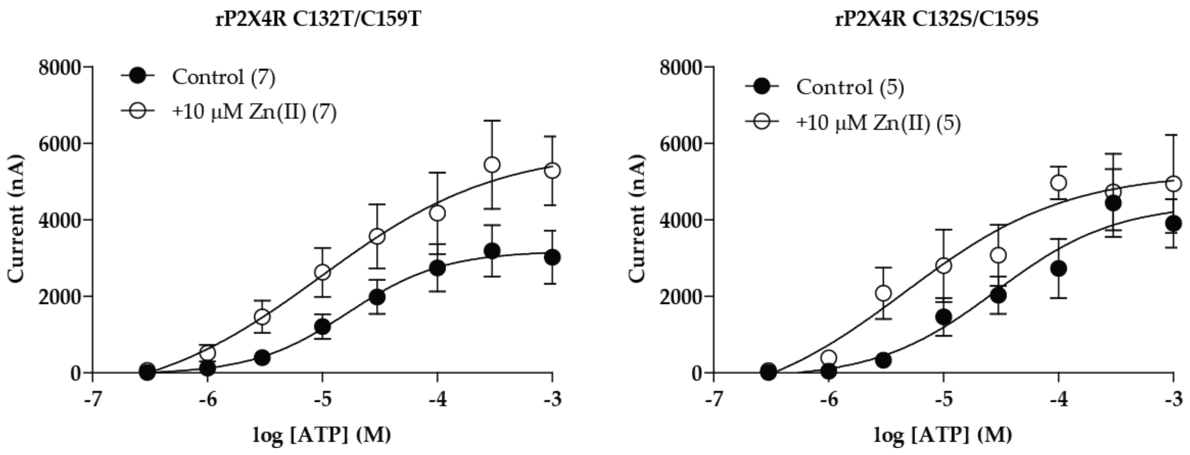

Figure S4. rP2X4 C132T/C159T and C132S/C159S double mutants are modulated by Zn(II). Concentration-response curves for the double mutants C132T/C159T (**left**) and C132S/C159S (**right**) in absence (black circles) and presence (white circles) of 10 μM Zn(II).

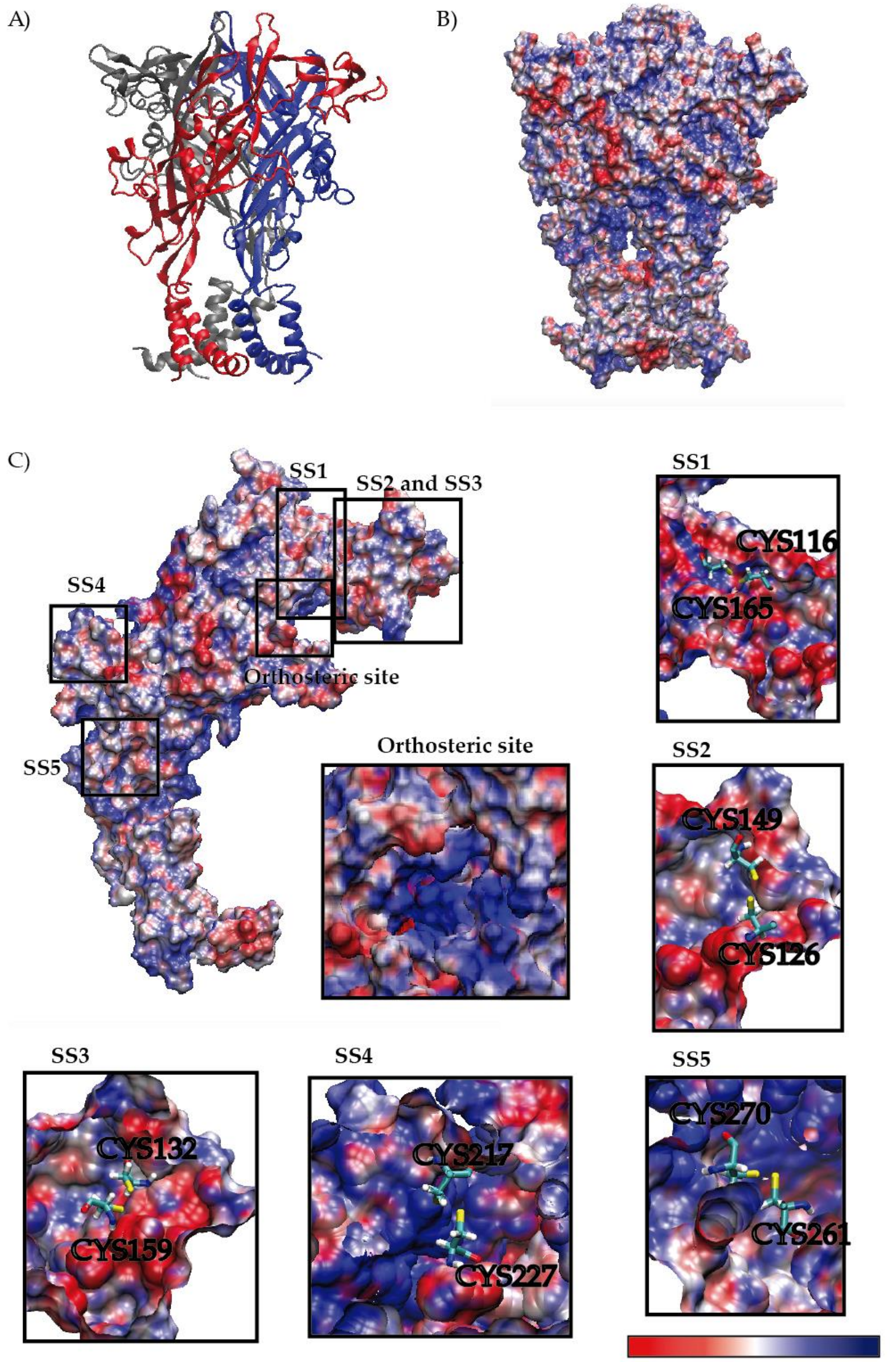

Figure S5. Electrostatic potential of the rP2X4R. Upper part (**A**) shows topological representation of the rP2X4R model head receptor domain and (**B**) the calculated electrostatic potential. (**C**) electrostatic potential representation in a rP2X4R monomer with magnifications of SS1-5 and orthosteric site. The color-coded electrostatic potential derived from the Poisson-Boltzmann solver APBS was graded from −5 kTe to 5.
